# A Landscape Study on COVID-19 Immunity at the Single-Cell Level

**DOI:** 10.3389/fimmu.2022.918383

**Published:** 2022-07-15

**Authors:** Rongguo Wei, Zheng Qin, Qi Huang, Lulu Liu, Fang Cheng, Songdong Meng, Lin Wang

**Affiliations:** ^1^ CAS Key Laboratory of Pathogenic Microbiology and Immunology, Center for Biosafety Mega-Science, Institute of Microbiology, Chinese Academy of Sciences, Beijing, China; ^2^ Savaid Medical School, University of Chinese Academy of Sciences, Beijing, China; ^3^ Department of Clinical Laboratory, The Fifth Affiliated Hospital of Guangxi Medical University, Nanning, China; ^4^ Department of Clinical Laboratory, The First People’s Hospital of Nanning, Nanning, China

**Keywords:** COVID-19, immunity, single-cell, signaling pathway, cytokines

## Abstract

Since 2019, the coronavirus (COVID-19) has outbroken continuously, spreading internationally and threatening the public health. However, it was unknown how the disorder at the single-cell level was associated with the pathogenesis of COVID-19. This study presented the disorders of macrophages, epithelial cells, CD8^+^ T cells, and natural killer (NK) cells at the single-cell level in the courses of COVID-19 and analyzed the immune response to cytokine storm. Compared with the healthy group, patients with COVID-19 had higher proportions of macrophages and lower proportions of T and NK cells, especially proportions of macrophages and epithelial cells with an increase during patients’ conditions from mild to severe. This study suggested that there were high levels of pro-inflammatory and chemokine expressions in cells of COVID-19 and analyzed cell subsets to explore its changes and pathways. It was worth noting that several subsets of macrophages, epithelial cells, CD8 T cells, and NK cells were involved in inflammation pathways, including interleukin-17 (IL-17) signaling pathway and tumor necrosis factor (TNF) signaling pathway. Moreover, the pathways interacting COVID-19 and cytokine receptor with each other were remarkably enriched. In addition, these cell subsets played important roles in inflammation, and their abnormal functions may cause COVID-19. In conclusion, this study provided an immune outlook for COVID-19 at the single-cell level and revealed different pathways in immune response of COVID-19 single cells.

## Introduction

The coronavirus disease 2019 (COVID-19) caused by severe acute respiratory syndrome coronavirus 2 (SARS-CoV-2), is the latest global pandemic with an increasing mortality (1, 2) and was described by the World Health Organization (WHO) as a public health emergency of global concern due to its widespread and rapid spread (3). This catastrophe has swept through countries around the world. Unfortunately, up to now, there are tens of thousands of cases being confirmed per day in several regions. The epidemic threatened to the public health and impacted social life and global economy (4). Therefore, it is urgent to explore the mechanism of immune disorders of COVID-19, having an essential part in the development of therapies and the diagnostic and preventive strategies. Studies have shown that cytokine storms, key factors in the prognosis of COVID-19, correlated with the severity of COVID-19 (5, 6).

Cytokine storm refers that, based on the microbial infection, body fluids of patients generate massive cytokines rapidly, such as TNF-α, IL-1, IL-6, IL-12, Interferon-α (IFN-α), IFN-β, IFN-γ, monocyte chemotactic protein-1 (MCP-1) and IL-8, resulting in the acute respiratory distress syndrome and multiple-organ dysfunction syndrome (7, 8). In addition, organs and tissue damage by COVID-19 is associated with systemic inflammation and so-called “cytokine release syndrome”. Although the coronavirus damages to epithelial (EP) tissue directly by injury and necrosis, evidence proved that the activation and disturbance of immune system were major factors of organ and tissue damage (9). According to studies in patients with COVID-19, viruses triggered the release of inflammatory mediators, mainly including inflammatory cytokines produced by monocytes and macrophages (5, 10). Extensive cell infiltration was observed in the patients’ lungs after death, dominated by macrophages (11). Moreover, many patients with COVID-19 reduced their T cells, particularly CD8+ T cells in critical patients (12, 13). SARS-CoV-2 studies have shown that the robust expression of pro-inflammatory factors was dominant among all patients with COVID-19 (14). Therefore, it is vital for the control of COVID-19 to elucidate why the inflammatory factors release and induct the cytokine storm.

Single-cell analysis, a valuable method, has been used to elucidate the phenotypes and expression differences among cell subsets in the peripheral immune responses associated with COVID-19 infection (15, 16). On the basis of multiple data, this study explored the immune landscape of patients with COVID-19 at the single-cell level with varying degrees, with a particular focus on cytokine storms. Furthermore, this study combed out the understanding of particular cell subsets, leading to the different therapies of patients with COVID-19, and expanded knowledge of these cell subsets of COVID-19 to identify interventions for COVID-19 in all probability.

## Materials and methods

### Data Source

Single-cell data sets of COVID-19 were collected from GEO database (https://www.ncbi.nlm.nih.gov/geo/). Demographic information and clinical data are shown in **Table S1**. GSE158055 (17), based on GPL24676 platform, totally gathered plenty of tissue samples from 196 individuals (n = 284), including peripheral blood mononuclear cells (PBMCs), bronchoalveolar lavage fluid, pleural effusion, and sputum. According to the WHO guidelines (https://www.who.int/publications/i/item/WHO-2019-nCoV-Surveillance_Case_Definition-2020.2), this study further categorized the COVID-19 samples into moderate recovery group, moderate progression group, severe recovery group, and severe progression group based on disease severity (moderate or severe) and staging (progression and recovery). In addition, it analyzed the cellular mechanisms only associating with the disease severity, and patients in the progressive and recovery phases were divided into severity groups. Thus, patients with mild and moderate symptoms were combined into a group (n = 122), patients with severe and critical symptoms (n = 134) in a group, and healthy people in a group (n = 28).

On the basis of the GPL24676 platform, GSE149689 (18) is a data set encompassing single-cell RNA sequences from 15 PBMC samples (asymptomatic, n = 1; mild, n = 4; severe, n = 6; and healthy, n = 4), including 11 patients with COVID-19 and four healthy people, of which five patients with influenza were not included in this study.

On the basis of the GPL23227 platform, GSE145926 (19) is a data set containing 12 single-cell sequencing samples, including nine patients with COVID-19 and three healthy people (severe, n = 6; mild, n = 3; and healthy, n = 3). All data sets were analyzed by the single-cell RNA sequencing (scRNA-seq) in 10x Genomics.

### Data Processing in ScRNA-Seq

First, this study employed IntegrateData function in the Seurat package of R language to combine the single-cell expression spectrum of three data sets (20), followed by clustering cells based on scRNA-seq data using the Seurat package and visualizing clusters by the Unified Flow Approximation and Projection (UMAP) package (21). To define the clustered marker genes, this study merge clusters featuring similar marker genes into one cell type. The first clustering (resolution = 9) identified 18 cell types, including T cells, natural killer (NK) cells, B cells, macrophages, and EP cells.

### Macrophage and EP, T, and NK Cell Reintegration

Macrophages and EP, T, and NK cells were reintegrated and reclustered with the Seurat package, respectively. This study performed the second clustering for macrophages and EP, T, and NK cells to identify clusters of each cell type. The second clustering followed the same procedures as the first one with a resolution range of 1 to 7. Subsequently, the resulting cell types were annotated with cell subclusters based on known markers.

### Analysis of Dynamic Changes in the Composed Cell Types

It calculated the proportion of cell types in each individual to investigate the dynamic changes in the composed cell types. For the sake of comparison, it obtained the relative variation in the composed cell types among all healthy people. At the same time, for each disease group, it reached the relative variation of each cell type by dividing cell types’ fractions of patients by the proportion of healthy donors.

### Analysis of Different Genes’ Expression

It utilized MAST to analyze the different genes’ expression in Seurat (FindAllMarkers function) (22). Genes were significantly upregulated if the mean natural logarithmic folding change (logFC) surpassed 0.5 and adjusted p-value less than 0.01. Genes with lower logFC than −0.5 and adjusted p-value than 0.01 indicated that they had a significant downregulation.

### Functional Enrichment Analysis

This study conducted the sub-aggregation analysis for each cell type by Seurat to explore the biological function of disease-specific subsets. On the basis of the differently expressed genes, Kyoto Encyclopedia of Genes and Genomes (KEGG) condensed to analyze each group of cells according to the cluster dissector of R package (23). According to default parameters, values with logFC lower than 0.5 and adjusted p-value less than 0.01 were significant.

### Data Analysis and Statistics

Data in the study were analyzed on the basis of Bioinforcloud (http://www.bioinforcloud.org.cn).

## Results

### Single-Cell Mapping of COVID-19 in a Large Scale

**Figure 1:**
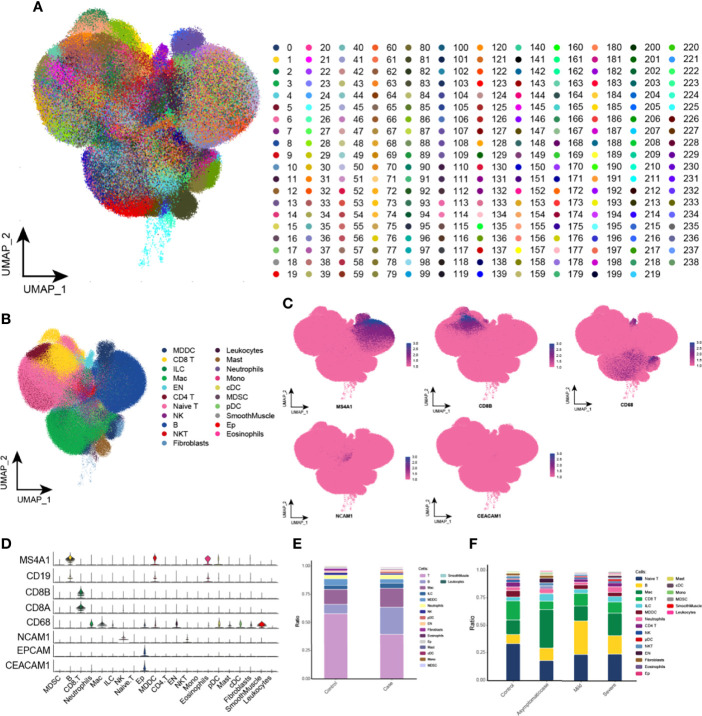
Global single-cell spectrum of COVID-19. **(A)** Global single-cell spectrum of COVID-19, with a total of 1,529,428 cells and 239 clusters obtained. **(B)** A total of 239 clusters were divided into different cell types based on known marker genes. **(C, D)** clusters of cells with marker genes mapped in the single-cell spectrum of CD8 T cells (CD8A and CD8B), macrophages (CD68), NK cells (NCAM1), EP cells (EPCAM1 and CEACAM1), and B cells (MS4A1 and CD19). **(E)** Cytobiological components and changes in the healthy and COVID-19 groups. **(F)** Cytobiological components and changes in the healthy group and patients with COVID-19 with different courses (asymptomatic, mild, and severe). EP cell, epithelial cell; KEGG, Kyoto Encyclopedia of Genes and Genomes; NK cell, natural killer cell; UMAP, Uniform Manifold Approximation and Projection for Dimension Reduction.

A total of 1,529,428 cells were obtained, for which this study established an expression matrix by gene count and reduced the dimensionality by UMAP and graph-based clustering to construct a single-cell atlas and identify 239 clusters (**Figure 1A**). On the basis of the marker genes, cell clusters were defined as 18 cell types (**Figure 1B**), mainly including T cells, macrophages, B cells, NK cells, and EP cells. In addition, these defined cell clusters expressed the corresponding cell markers (**Figures 1C**, **D**). Subsequently, this study explored the changes in the proportion of cell types of different progression between healthy and COVID-19 groups and obtained higher proportions of B cells and macrophages and lower proportions of T cells and NK cells in patients with COVID-19 compared with the healthy group (**Figure 1E**). Whereas, compared with patients with mild disease, the severe patients contained higher proportions of macrophages and EP cells and lower proportions of B cells (**Figure 1F**). Moreover, T cell abundance was reduced in the bodies of patients with COVID-19, most likely because patients were susceptible to be infected under the condition of T cell depletion, revealing that COVID-19 immune cells were diverse with different proportions in mild and severe cases.

### Abnormal Components of Macrophage in Patients with COVID-19

**Figure 2:**
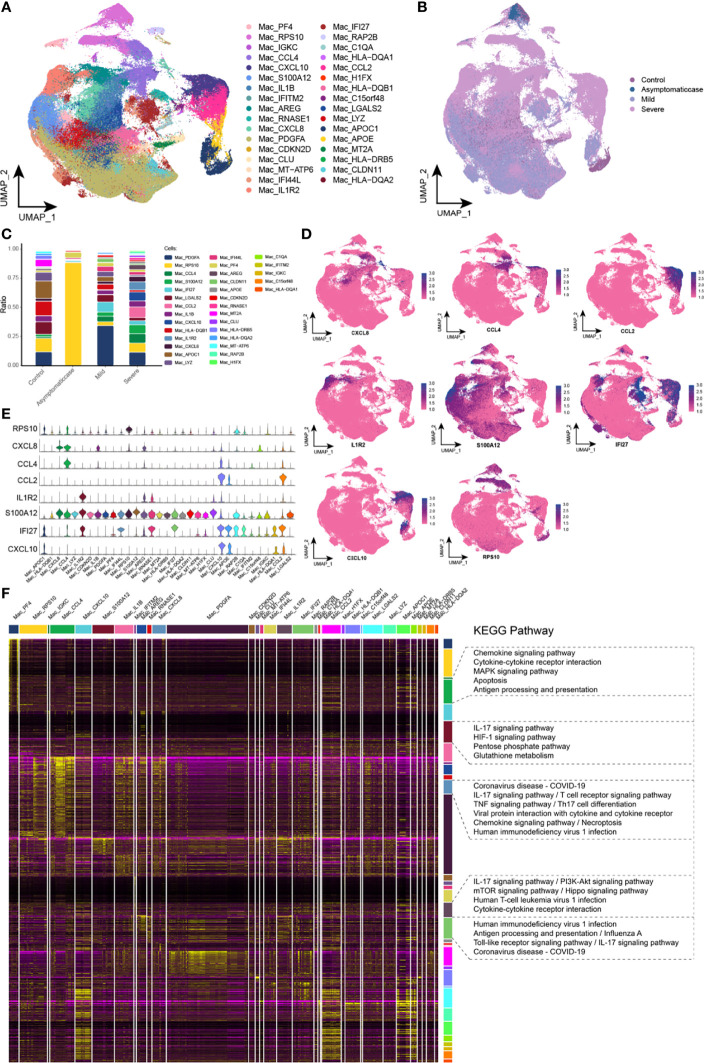
Abnormal components of macrophage in patients with COVID-19. **(A)** Single-cell mapping of subsets of macrophages. **(B)** Single-cell mapping of macrophages mapped in the COVID-19 courses. **(C)** Components and changes of macrophage subsets in the COVID-19 courses. **(D, E)** Expression of marker genes in macrophage subsets. **(F)** Biological pathways involved in macrophage subsets with specific changes. KEGG, Kyoto Encyclopedia of Genes and Genomes; Mac, macrophage; UMAP, Uniform Manifold Approximation and Projection for Dimension Reduction.

Initially, proportion of macrophages scaled up in patients with COVID-19. For the sake of heterogeneity of macrophages during the different courses, this study reclustered macrophages and obtained 33 clusters totally (**Figure 2A**). In addition, the abundance of macrophage subpopulations in different disease courses was mapped in the single-cell atlas (**Figure 2B**). This study provided the variation analyses of macrophage components during the different courses, and, specifically, compared with the control group, there was a big difference of cell components among asymptomatic, mild, and severe patients, reflected in the corresponding subsets (**Figure 2C**). Whereas, compared with the other, the asymptomatic group had a significant increase of RPS10 abundance. In addition to those, compared with the control and asymptomatic groups, several subsets, such as CCL4, S100A12, IFI27, CCL2, CXCL10, IL1R2, and CXCL8, presented their specific expressions, and, particularly, CCL2, CXCL10, and IL1R2 were specifically expressed in the severe group. Single-cell spectrum of marker genes’ subsets was shown in **Figure 2D**, and marker genes were highly expressed in specific subsets (**Figure 2E**). According to data, the homeostasis of macrophage sets in patients with COVID-19 was not adjusted, and genes and chemokine expressions in the specific subsets were associated with inflammation. In addition, cell concentration revealed that these specific subsets were mainly involved in some inflammatory pathways, such as coronavirus of COVID-19, IL-17 signaling pathway, chemokine signaling pathway, TNF signaling pathway, and viral protein interaction between cytokine and its receptor (**Figure 2F**). Therefore, it could be hypothesized that several subpopulations of macrophages were highly expressed in various cytokines, especially higher expression in the severe patients that maybe an origin of cytokine storm.

### Abnormal Components of EP Cells in Patients with COVID-19

**Figure 3:**
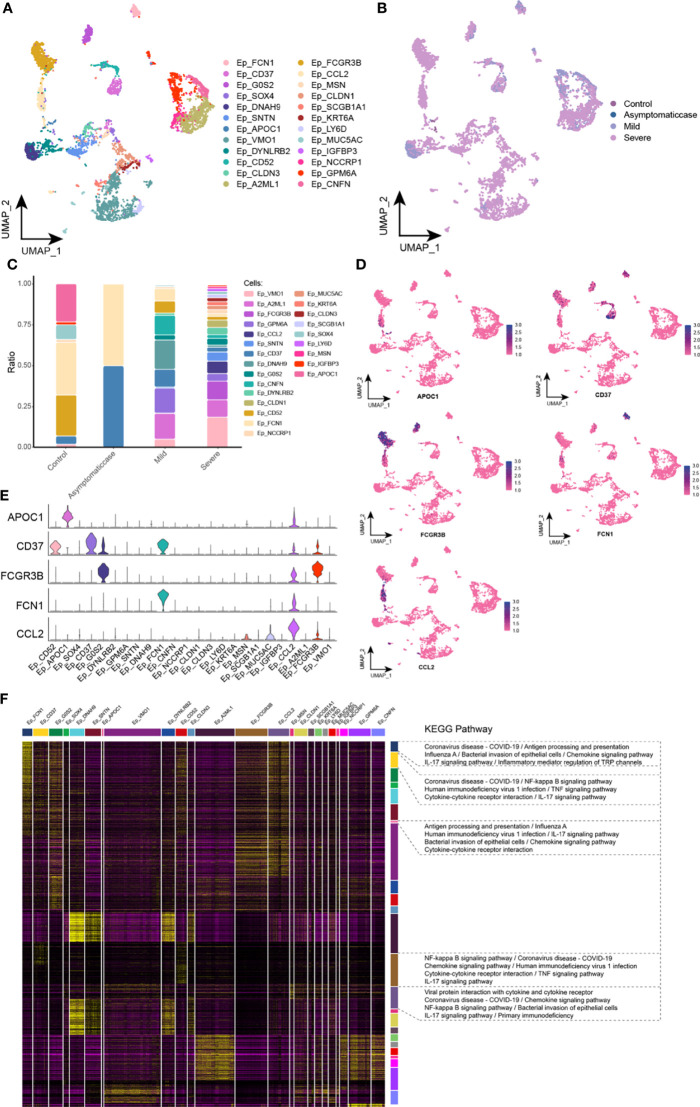
Abnormal components of epithelial cell in patients with COVID-19. **(A)** Single-cell mapping of EP cell subsets. **(B)** Single-cell mapping of EP cells in the COVID-19 courses. **(C)** Components and changes of epithelial cell subsets in the courses of new crowns. **(D, E)** Expression of marker genes in subsets of EP cells. **(F)** Biological pathways involved in subsets of EP cells with specific changes. EP, epithelial cell; KEGG, Kyoto Encyclopedia of Genes and Genomes; UMAP, Uniform Manifold Approximation and Projection for Dimension Reduction.

This study reclustered EP cells and obtained 24 clusters in total (**Figure 3A**). In UMAP, projections of EP cells show heterogeneity in mild and severe cases (**Figure 3B**). A big difference among clusters could be given in COVID-19 courses (**Figure 3C**). In detail, different from critical patients, CD37 and Ficolin 1 (FCN1) abundance was increased remarkably in the asymptomatic and healthy groups, and CCL2 was specifically expressed in the critical patients. Apolipoprotein C1 (APOC1) had a low level in the critical patients, suggesting that APOC1 was associated with the severity of COVID-19. The single-cell spectrum of marker genes was shown in **Figure 3D**, and they have high expression in specific subsets (**Figure 3E**). In summary, data suggested that the homeostasis of EP cells in patients with COVID-19 was not adjusted and that specific subset factors were associated with inflammation and immune responses. On the basis of preconcentration analysis of cells, specific subsets were mainly involved in inflammation pathways, containing coronavirus of COVID-19, influenza A, human immunodeficiency virus type 1, interaction between cytokine and its receptor, bacterial invasion of EP cells, and chemokine signaling pathway (**Figure 3F**).

### Accommodative Dysfunction of CD8 T Cells Under the COVID-19 Courses

**Figure 4:**
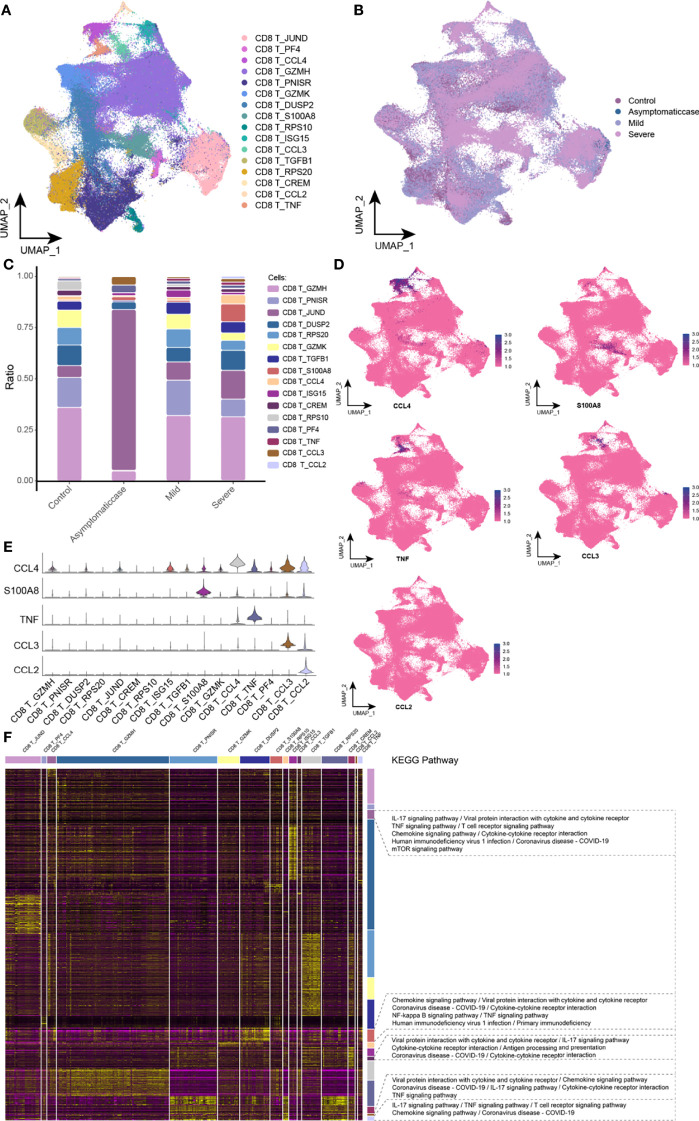
Abnormal components of CD8 T cell in patients with COVID-19. **(A)** Single-cell mapping of CD8 T cell subsets. **(B)** Single-cell mapping of CD8 T cells in the COVID-19 courses. **(C)** Components and changes in CD8 T cell subsets in the COVID-19 courses. **(D, E)** Expression of marker genes in CD8 T cell subsets. **(F)** CD8 T cells with specific changes in subsets involved in biological pathways. KEGG, Kyoto Encyclopedia of Genes and Genomes; UMAP, Uniform Manifold Approximation and Projection for Dimension Reduction.

This study divided CD8 T cells into 16 branches (**Figure 4A**) and mapped in the COVID-19 courses (**Figure 4B**). It was worth noting that, in the critical group, specific expression of S100A8, CCL4, TNF, CCL3, and CCL2 subsets was presented (**Figure 4C**). Excessive cytokine expression resulted in an excessive inflammation response that may be a key factor of cytokine storm. The single-cell spectrum of marker genes in subsets was given in **Figure 4D**, and they had high expression in specific subsets (**Figure 4E**). On the basis of preconcentration analysis of cells, specific subsets were mainly involved in certain inflammations and pathways (**Figure 4F**), such as IL-17 signaling pathway, TNF signaling pathway, T cell receptor signaling pathway, the interaction between cytokine and its receptor, and the interaction of coronavirus of COVID-19 and its cytokine receptor. Ultimately, this study hypothesized that SARS-CoV-2 affected the release of cytokine and therefore T cell depletion.

### Accommodative Dysfunction of NK Cells Under the COVID-19 Courses

**Figure 5:**
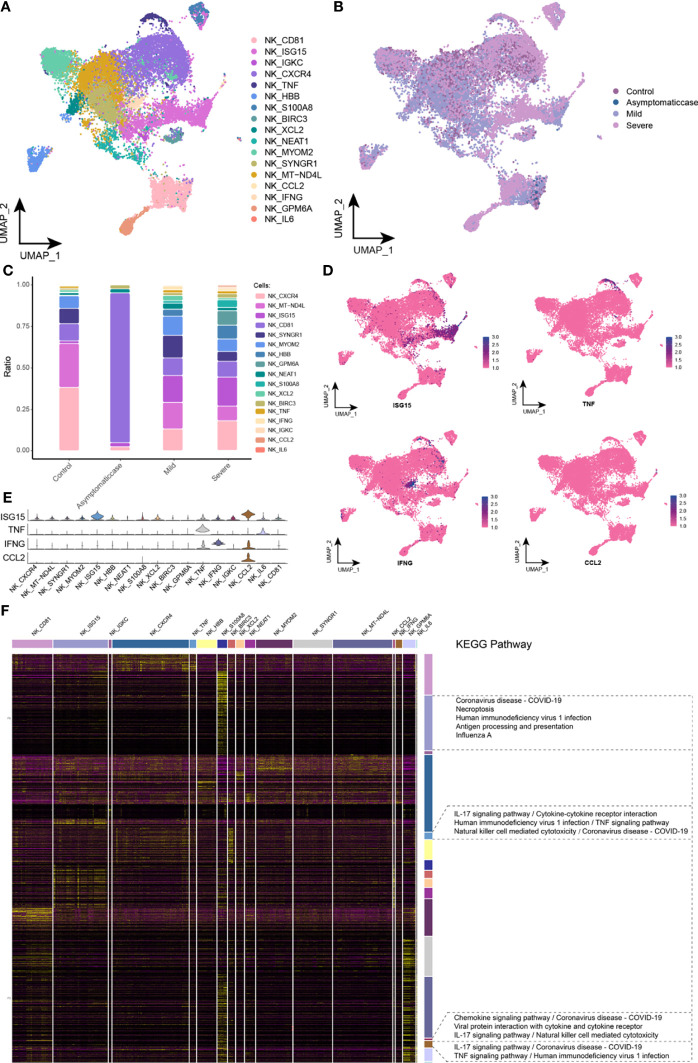
Abnormal components of NK cell in patients with COVID-19. **(A)** Single-cell mapping of NK cell subsets. **(B)** Single-cell mapping of NK cells in the COVID-19 courses. **(C)** Components and changes of subpopulations of NK cells in the courses of new crowns. **(D, E)** Expression of marker genes in NK cell subsets. **(F)** Biological pathways involved in NK cell subsets with specific changes. KEGG, Kyoto Encyclopedia of Genes and Genomes; NK, natural killer cell; UMAP, Uniform Manifold Approximation and Projection for Dimension Reduction.

After reclustered NK cells, this study gained a total of 17 clusters (**Figure 5A**) and mapped in the COVID-19 courses (**Figure 5B**). Specifically, compared with the healthy and asymptomatic groups, NK cell subsets gathering under the COVID-19 courses referred to the significant difference of cytobiological factors in the mild and severe groups (**Figure 5C**). Compared with the healthy group, ISG15 subsets performed an upregulation in mild and severe patients, whereas the asymptomatic cases were not concentrated to ISG15 subsets. ISG15 is an interferon-stimulating gene (ISG), which was usually associated with viral RNA perception (24). Results suggested that the intensity of interferon response may be relevant to the disease severity of patients with COVID-19. Moreover, few chemokines, such as TNF, IFNG, and CCL2 subsets, were expressed in the mild and severe groups. The single-cell spectrum of marker genes for subsets was given in **Figure 5D**, and they had high expression in the certain subsets (**Figure 5E**). On the basis of preconcentration analysis of cells, these subsets were mainly involved in the new crown pneumonia and inflammation pathways containing TNF signaling pathway, coronavirus of COVID-19, IL-17 signaling pathway, NK cell–mediated cytotoxicity, and TNF signaling pathway (**Figure 5F**). Thus, this study further hypothesized that, in the late stage of COVID-19, NK cells could express various pro-inflammatory factors that mediated the antiviral response of bodies.

## Discussion

Patients with severe COVID-19 mainly present with suffering from the respiratory tract diseases, but as the disease goes, it may lead to critical pulmonary diseases and relevant complications with high incidence and mortality (25). Currently, COVID-19 is still raging around the world, but there is no optimal treatment or effective drug for this fatal lung disease. In this study, we integrated single-cell sequencing data from three data sets (GSE158055, GSE149689, and GSE145926) and analyzed for patients with COVID-19 under different courses to construct a global landscape of single cells of COVID-19. Furthermore, on the basis of the analyses of macrophages, EP cells, CD8 T cells, and NK cells with significant changes under the COVID-19 courses, marked inflammation factors and pathways participated in the cell subsets. People with SARS-CoV-2 tended to present the immune response and inflammation in their lungs, release the inflammation of cytokines and chemokines, and make further responses (5).

In addition, compared with the control and asymptomatic individuals, few subsets, including CCL4, S100A12, IFI27, CCL2, CXCL10, IL1R2, and CXCL8, were specifically expressed in the mild and severe groups. Macrophages that distribute the whole body have essential parts in tissue homeostasis. In the inflammation processes, tissue-resident macrophages and infiltrating monocyte were motivated by the tissue microenvironment and additional layers caused by disease-related factors, immune cells, soluble infiltration, and, sometimes, pathogenic microorganisms (26). Studies suggested that the increase of circulating levels in the pro-inflammatory cytokines (such as the interleukin IL-1B, IL-6, and IL-12) and chemokines (CXCL10 and CCL2) has been associated with lung inflammation and lesion of patients with SARS, which was similar to those of patients with MERS-CoV (27–29). In addition, patients with COVID-19 also presented the high level of pro-inflammatory factors and chemokines (5). However, recent studies have shown that chemokines, playing key roles in COVID-19, are relevant to cytokine storm of patients (30). Results showed that compared with patients with less severe infections without Intensive Care Unit (ICU), patients with COVID-19 with ICU had higher concentrations of CXCL10, CCL2, and TNF-α (5). Cytokine storm could easily attack the immune system, leading to adult respiratory distress syndrome (ARDS), multi-organ failure, and even death (9). Moreover, the so-called cytokine storm contributed to enter into a more severe course. The interferon, interleukin, chemokines, and TNF were main factors of the development of cytokine storm for patients with COVID-19. This study found a significant high expression of FCN1 of EP cells in the asymptomatic and healthy individuals. FCN1 was apt to assist and improve the inflammatory response of organism (31), which explained why asymptomatic patients had the mild or insignificant clinical symptoms. Furthermore, FCNR3B was specifically expressed in critical patients, and the immunoglobulin Fc receptor regulated the adaptive and innate immune responses, which was essential against infection and prevention of chronic inflammation and autoimmune diseases (32). NK and T cells were the immune effector cells against virus infection (33). T cell responses in the early stage had essential roles in the removal of viruses in the acute respiratory infection process. In addition, cytokine storm may affect the severity of coronavirus by reducing the number of T cells (34). In this study, CD8 T cells expressed its cytokines excessively, causing excessive inflammatory response, which may be the key cause of cytokine storm. Meanwhile, high expression of ISG15 in NK cells, as an interferon-stimulating factor (ISG), was commonly associated with viral RNA perception (35) of critical patients, and our results suggested that the intensity of interferon response was related to the severity of patients with COVID-19. Furthermore, under the preconcentration of cell subsets expressing specifically, the signaling pathways characterized by plenty of inflammation pathways, viral infection pathways, and antigen presentation-promoting functions were enriched.

In conclusion, this study constructed a new large-scale single-cell landscape of COVID-19 through analyzing data of single cells from large cohorts of COVID-19. In addition, on the basis of the further analysis of immune cells, it explored the potential sources of cytokine storms. However, it had some limitations. First, it explored changes in single-cell components of patients under the COVID-19 courses. Second, it did not establish the connection among cells. In the future, we will further explore the differences in the progression stages, age stages and samples, as well as the connection between cells’ communication.

## Data Availability Statement

The datasets presented in this study can be found in online repositories. The names of the repository/repositories and accession number(s) can be found in the article/**Supplementary Material**.

## Author Contributions

RW, ZQ, and SM participated in the study conceptualization and design of experiments. RW, ZQ, QH, LL, and FC collected data, conducted the data analysis and interpretation of results. RW, ZQ, QH, LL, and LW performed experiments. RW, SM, and LW wrote and edited the original draft. All authors contributed to the article and approved the submitted version.

## Funding

This work was supported by a grant from the Strategic Priority Research Program of the Chinese Academy of Sciences (XDB29040000), the Guangxi Medical and Health Key Discipline Construction Project (Department of Clinical Laboratory), the Guangxi Zhuang Autonomous Region Health and Family Planning Commission Self-financed Scientific Research Project (No. Z20200208), and the Project of Nanning Scientific Research and Technology Development Plan (20193252).

## Conflict of Interest

The authors declare that the research was conducted in the absence of any commercial or financial relationships that could be construed as a potential conflict of interest.

## Publisher’s Note

All claims expressed in this article are solely those of the authors and do not necessarily represent those of their affiliated organizations, or those of the publisher, the editors and the reviewers. Any product that may be evaluated in this article, or claim that may be made by its manufacturer, is not guaranteed or endorsed by the publisher.

## Supplementary Material

Supplementary Table 1 Clinical data of the enrolled COVID-19 patients examined by scRNA-seq.
